# Fetal‐haemoglobin enhancing genotype at *BCL11A* reduces HbA_2_ levels in patients with sickle cell anaemia

**DOI:** 10.1002/jha2.186

**Published:** 2021-05-04

**Authors:** Titilope A. Adeyemo, Oyesola O. Ojewunmi, Idayat Ajoke Oyetunji, Olufunto Olufela Kalejaiye, Stephan Menzel

**Affiliations:** ^1^ Department of Haematology and Blood Transfusion, College of Medicine University of Lagos Idi‐Araba Lagos Nigeria; ^2^ Sickle Cell Foundation Nigeria Idi‐Araba Lagos Nigeria; ^3^ Haematology/Oncology Unit, Department of Medicine, College of Medicine University of Lagos Idi‐Araba Lagos Nigeria; ^4^ School of Cancer and Pharmaceutical Sciences King's College London London UK

**Keywords:** African patient population, BCL11A, genetic analysis, HbA, sickle cell disease

## Abstract

Understanding the interplay of genetic factors with haemoglobin expression and pathological processes in sickle cell disease is important for pharmacological and gene‐therapeutic interventions. In our nascent study cohort of Nigerian patients, we found that three major disease‐modifying factors, HbF levels, α‐thalassaemia deletion and *BCL11A* genotype, had expected beneficial haematological effects. A key *BCL11A* variant, while improving HbF levels (5.7%–9.0%), also led to a small, but significant decrease in HbA_2_. We conclude that in general, interventions boosting HbF are likely to reduce HbA_2_ in patients’ erythroid cells and that such therapeutic strategies might benefit from a parallel stimulation of HbA_2_ through independent mechanisms.

## 1 BACKGROUND

Sickle cell anaemia (SCA, homozygosity of the β^S^ globin mutation), one of the commonest severe genetic disorders, is most prevalent in Africa. Every year, 150,000 affected children are estimated to be born in Nigeria alone, an ‘epicentre’ of this debilitating disease. Insight into the causes of variable severity, which is strongly influenced by patients’ genetic background, has stimulated the development of new therapeutic approaches. Attention is presently focused on fetal haemoglobin (HbF, α_2_γ_2_) persistence as an alleviating factor, but also a raised presence of the minor adult haemoglobin, HbA_2_ (α_2_δ_2_), can inhibit HbS polymerisation [1, 2], the key pathological mechanism.

In general, HbA_2_ levels in a person are altered when the production of the major adult globin chains is impaired. For instance, in carriers of α thalassaemia mutations, they are decreased due to enhanced competition of the δ chain with the β chain, which has a higher affinity for pairing with the rate‐limiting α chains. In the presence of β thalassaemia mutations [3] and β globin gene (*HBB*) regulatory variants [4], on the other hand, HbA_2_% is increased because of reduced β chain competition. Genetic variation at the HbF modifier locus *HBS1L‐MYB* was found to also boost HbA_2_ levels in non‐anaemic subjects, while HbF modifier variants at the *BCL11A* and *Xmn1‐HBG2* loci had no such effect [4]. In sickle cell disease however, due to the effects of the mutation on the properties of the β chain, the relationship between HbF and HbA_2_ levels, and with it the effect of genetic HbF modifiers on HbA_2_ levels, is fundamentally altered [5]. The defective β globin chain, β^S^ (forming HbS), has reduced affinity to the α chain, compared not only to the native β chain, but also to the γ and δ chains. In this situation, any increase in γ globin presence will lead to the replacement of a weaker competitor (β^S^) with a stronger one (γ), leading to a reduction of δ chain inclusion into haemoglobin and to a reduction in HbA_2_ levels. Therefore, in contrast to the direct correlation we observed in non‐anaemic adults [4], levels for HbF% and HbA_2_% appear to have an inverse, or antagonistic, relationship in Hb SS erythrocytes [6]. A study of American SCD patients [5] detected a significant influence of *HBS1L‐MYB* and *HBB* loci on HbA_2_ levels, but also of the *BCL11A* locus, which occurred indirectly, mediated through its effect on HbF levels.

Dissecting the interplay of genetic factors with various haemoglobin species and disease phenotype in sickle cell disease will help to better understand, predict and adjust the effects of pharmacologic and gene therapeutic intervention into this complex system.

We investigated the influence of three major disease‐modifying factors, HbF levels, deletional α thalassaemia and *BCL11A* genotype, on haematological, biochemical and some clinical parameters in a group of 244 Nigerian patients with sickle cell anaemia. As a representative marker for this locus, we have chosen *rs1427407*, which tags its main HbF ‐increasing allele [7, 8].

## 2 PATIENTS AND METHODS

Two hundred and forty‐four patients with sickle cell anaemia (HbS homozygous), 5–46 years of age, neither on hydroxyurea nor transfusion, were recruited at the Lagos University Teaching Hospital (approved as ADM/DCST/HREC/1686) as part of a cross‐sectional genetic study that has been described previously [9]. Our patient population is characterised by the absence of genetic HbF modifier *XmnI‐HBG2* (Senegal or Arab‐Indian β‐globin haplotypes) and by the presence of thalassaemia deletions (‐α^3.7^) in 33% of patients.

Genotyping for *rs1427407* (TaqMan assay) was carried out as previously described [9], and the ‐α^3.7^ thalassaemia deletion was assayed by multiplex PCR (polymerase chain reaction) [10]. Cation exchange high performance liquid chromatography (D‐10 HPLC System, Bio‐Rad Laboratories, Hercules, CA, USA) was used to confirm SCA diagnosis and to measure HbF and HbA_2_ levels. Patients with Hb SC and Hb S/β^+^thal genotypes were excluded through this approach, whereas an expected small number of Hb S/β^0^thal patients (<1% in our population) was retained. Such patients do not produce HbA and are, in effect, clinically and pathogenetically SCA. HbA_2_ levels measured by HPLC are overestimated in the presence of HbS [11], but we do not expect this to systematically skew our findings, such as HbA_2_ level differences between patient groups. Full blood count was determined by Mindray BC‐2800 (China) and reticulocyte count as described previously [12].

## 3 RESULTS AND DISCUSSION

The effects on haematological profiles of our SCA patients were recorded for the three major disease‐modifying factors HbF, α thalassaemia and *BCL11A* genotype (Table 1).

**TABLE 1:** Influence of three major disease‐modifying factors on haematological parameters in patients with sickle cell anaemia

	α thalassaemia genotype			*BCL11A* (*rs1427407*) genotype			HbF (*n* = 244)	
	αα/αα (*n* = 112)	α‐/αα (*n* = 49) or α‐/α‐ (*n* = 6)	*p*‐value	GG (*n* = 138)	GT (*n* = 98) orTT (*n* = 8)	*p*‐value	β*	*p*‐value
Hb° (g/dL)	8.2 (7.6–9.1)	8.5 (7.8–9.1)	0.344	8.2 (7.6–8.8)	8.6 (7.8 –9.1)	**0.009**	0.125	**<0.0001**
PCV°	24.6 (21.9–27.2)	25.8 (24–27.7)	0.037	24.9 (22.2–27.0)	25.1 (23.3–27.6)	0.052	0.003	0.43
RBC° (x10^12^/L)	2.9 (2.6–3.1)	3.3 (2.9–3.7)	**<0.0001**	3.0 (2.6–3.4)	3.0 (2.7–3.3)	0.324	‐0.04	0.57
MCV° (fL)	85.7 (81.8–91.2)	77.5 (72.1–83.3)	**<0.0001**	83.2 (76.6–87.7)	84.4 (80.4–90.6)	0.076	0.034	**<0.0001**
MCH^§^ (pg)	29.2 (2.9)	25.7 (3.0)	**<0.0001**	27.3 (3.5)	28.1 (3.3)	0.066	0.07	**<0.0001**
MCHC° (g/dL)	33.7 (32.8–34.7)	32.8 (31.6–33.9)	**<0.0001**	33.3 (32.2–34.2)	33.0 (32.4–34.2)	0.617	‐0.04	0.20
Leukocytes^§^ (x10^9^/L)	12.1 (3.6)	11.4 (3.7)	0.234	11.8 (9.8 –14.6)	11.7 (9.0–14.7)	0.178	‐ 0.053	**<0.0001**
Platelets° (x10^9^/L)	430 (341–537.5)	406 (310–492)	0.092	444 (352–540)	430.0 (340.0–494.0)	0.036	0.00	0.09
Reticulocytes^§^ (%)	9.7 (3.8) *n* = 103	8.7 (3.9); *n* = 45	0.139	9.4 (3.8)	8.3 (3.7)	0.024	‐0.01	0.49
HbF° (%)	6.3 (3.6–9.6)	5.3 (3.0–8.1)	0.253	5.7 (3.7)	9.0 (4.7)	**<0.0001**	–	–
HbA_2_^§^ (%)	3.6 (0.5)	4.2 (0.6)	**<0.0001**	4.0 (0.6)	3.6 (0.6)	**<0.0001**	**‐0.611**	**<0.0001**

Results are presented as mean (standard deviation) for data that were normally‐distributed (groups compared by Student's *t*‐test^§^), while those that were not are presented as median (interquartile range) and compared by Mann‐Whitney U test°.

*HbF: Univariate linear regression was performed using age and sex as covariates, the regression coefficient (β) indicating the direction of the effect of HbF on blood count and HbA_2_ values. The *p*‐values shown have not been corrected for multiple comparison.

Higher HbF levels were associated with improved blood parameters in the patients, that is, raised total haemoglobin and reduced leukocyte counts (Table 1). A previously reported [5, 6] depression of HbA_2_ in the presence of more HbF was also detected.

Co‐inheritance of the α‐thalassaemia deletion (‐α^3.7^), observed in 32.9% of patients (α‐ / αα: 29.3%, α‐ / α‐: 3.6%), led to a typical [13, 14] decrease of red blood cell size and haemoglobin content (Table 1). We did not detect the frequently reported [13] improved total haemoglobin or a reduction in haemolysis indicators (reticulocyte count, LDH, data not shown), but did measure a marked increase in red blood cell numbers. A tendency towards improved haematocrit (p = 0.037) is not significant when multiple testing is considered but is nevertheless in keeping with the disease‐alleviating character of alpha thalassaemia deletions.

Significantly elevated HbA_2_ levels were seen in α deletion‐carriers (Table 1), a paradoxical effect known for sickle cell disease, where δ chains have a competitive advantage over the low‐affinity β^S^ chain when α chains are in limited supply [15, 16].

*BCL11A* genotype affected HbF levels significantly, as expected. Presence of the minor allele (‘*T*’) of the key *BCL11A* enhancer variant *rs1427407* led to an improvement of anaemia (increased Hb levels, p = 0.009), as has been reported for other SCD patient populations [8, 17, 18] and also to a tendency towards decreased platelet (p = 0.036) and reticulocyte (p = 0.024) numbers. While patients carrying *rs1427407‐T* had higher average HbF levels – 9.0% of total haemoglobin, compared to 5.7% in patients lacking this allele (p < 0.0001) – they showed a decrease of the presence of HbA_2_ from 4.0% to 3.6% (p < 0.0001). The loss of HbA_2_ is relatively small, and an overall HbS‐diluting effect of this *BCL11A* enhancer variant is still evident. Nevertheless, our data appear to show that genetic (and possibly also pharmacological) alteration of HbF production in sickle cell disease is likely to be accompanied by some reduction in HbA_2_. HbF (heterocellular, i.e., restricted to F cells [19]) and HbA_2_ (pancellular [20]) have a different distribution pattern across the population of red blood cells of a patient. Present (hydroxyurea) and future treatment strategies that increase HbF and F cell release could be complemented with a therapeutic approach to boost HbA_2_ through an independent pathway, such as gene‐regulatory elements detected through HbA_2_‐associated genetic variants within the beta globin gene locus [4].

## AUTHOR CONTRIBUTIONS

Study design: Adeyemo TA, Ojewunmi OO and Menzel S. Conduct of research: Adeyemo TA, Ojewunmi OO and Oyetunji AI. Writing of the paper: Adeyemo TA, Ojewunmi OO and Menzel S. Analysis of data: Ojewunmi OO. Samples and clinical data: Kalejaiye OO. Contribution of essential patients: Kalejaiye OO.

## Data Availability

Summary data supporting the findings of this study are available upon reasonable request from the corresponding author. Individualised patient data cannot be shared due to privacy and ethical restrictions.
